# Effects and Risk Evaluation of Oil Spillage in the Sea Areas of Changxing Island

**DOI:** 10.3390/ijerph110808491

**Published:** 2014-08-19

**Authors:** Hanxi Wang, Jianling Xu, Wenkui Zhao, Jiquan Zhang

**Affiliations:** 1 School of Environment, Northeast Normal University, Changchun 130117, China; E-Mail: wanghanxizs1982@126.com; 2 National Marine Environmental Monitoring Center, Dalian, Liaoning 116023, China; E-Mail: wkzhao@nmemc.gov.cn.com

**Keywords:** oil spillage, effect on marine environment, risk evaluation, preventive measures

## Abstract

This paper evaluated the oil spillage risk in the waters near the island of Changxing in Dalian (China) based on the established risk assessment index. Four wind regimes (windless, northerly wind, westerly wind and southerly wind) were selected as weather conditions for the dynamic prediction of oil drift. If an oil spill occurs near the Koumen (a place near the island of Changxing), the forecast and evaluation are conducted based on a three-dimensional mathematical model of oil spillage, and the results obtained show the scope of the affected area when winds from various directions are applied. The oil spillage would, under various conditions, flow into the northern and western sea area of Changxing Island Bay, namely the Dalian harbor seal National Nature Reserve, and create adverse effects on the marine ecological environment. The rationality of combining the established oil spillage risk comprehensive index system with model prediction is further confirmed. Finally, preventive measures and quick fixes are presented in the case of accidental oil spillages. The most effective method to reduce environment risk is to adopt reasonable preventive measures and quick fixes.

## 1. Introduction

The risk of accidental oil spillages has increased in domestic and international waters in recent years [1,2,3,4,5]. For example, one of the worst accidents, the Gulf of Mexico oil spill, was the costliest in history to the world oil industry [1,3,4]. The statistics of the International Tanker Owners Pollution Federation (ITOPF) [6] show that a total of 7847 oil spillages occurred during 39 years, of which 79.5% of occurrences were less than 7 t, 15.9% were in the 7 to 700 t range, and 4.6% were greater than 700 t.

Aspects of tanker loading and unloading operations accounted for 40.2% of the total. There are four main types of accidents: refueling, collisions, groundings and hull damage, and these accident types account for 2.4% to 21.4% of the total. Since 79.5% of occurrences involved less than 7 t, this shows that the oil spill accidents are mainly small; collision and handling oil spillages occurred in the 7–700 t range. Massive oil spillage occurrences in the past 38 years have been minimal, and there is a trend that large-scale oil spillage accidents greater than 700 t will be significantly reduced in number.

From 1973 to 2006, oil spills from ships occurred approximately 2635 times, including oil spillages of more than 50 t from ships in China’s 69 coastal areas. A total spill volume of 37,077 t occurs twice a year on average, with approximately 537 t of oil spillage pollution [7].

Oil spillages cause serious harm because of the way they spread. For example, they can pollute the marine environment, affect fish survival, destroy the ecological balance of the marine ecosystem and harm human health [8]. Analysis of the environmental impacts caused by oil spillages on the surrounding waters, using risk prediction and evaluation, can provide references for offshore oil production, transportation security and management to reduce the environmental risks.

The United States Environmental Protection Agency (EPA) proposed the concept of ecological risk assessments in 1990. Ecological Risk Assessment theories and methods are at the international forefront of research in the United States [9]. In 1993, the EU issued regulations for ecological risk assessment of chemicals, created technical guidance documents and conducted a systematic ecological risk assessment of chemicals and broad industrial pollutants in Europe to explore the methods and procedures of different areas [10,11].

Currently, many scholars from various countries are focused on risk assessment, mainly concentrated on the analysis and evaluation of chemical accidents, health risks and natural disaster risks [12,13,14,15,16,17,18,19]. However, such analyses have not been conducted in marine oil spill risk assessment studies. This study uses Changxing Island in Dalian as an example to study dynamic predictions under different weather conditions. We conduct potential ecological risk analysis and evaluation according to a three-dimensional mathematical model to provide reference for the protection of the marine environment and oil spillage control.

## 2. Materials and Methods

### 2.1. Three-Dimensional Mathematical Model to Predict Marine Oil Spillage

Oil spillage in marine waters includes two main movement processes: the overall displacement under the effect of advection and shear flow and turbulent diffusion. The expansion of the oil spillage by itself is very short; longer durations exist mainly as a result of stratospheric transport and turbulent diffusion. We intend to adopt the “oil particle” method to simulate the space-time behavior of oil spillages in the marine environment. This method can faithfully to represent many characteristics of actual observations with respect to the spread of an oil spillage:

(1) The movement of spilled oil

According to the Lagrange viewpoint, the spatial displacement of individual particles in the time Δt can be expressed as:

(1) $$\Delta r_{i}=u_{i}\Delta t+w_{i}\Delta t+r_{i}^{\prime }$$

The vector *r_i_* represents the *i*th particle position and vector *u_i_* represents the time step size at the beginning of the particle position advection speed. The vector *w_i_* represents the vertical settling velocity for the *i*th particle. The random variable $r_{i}^{\prime }$ is a random walk distance.

We used tidal current and wind current as the advection transport medium to run the synthesis of the geotropic flow and the synthesis of density flow. Advection caused by the displacement of each particle is simple to obtain:

(2) $$\begin{array}{l} D_{x}=u\cdot D_{t}\\ D_{y}=v\cdot D_{t} \end{array}$$

where *u* is the horizontal velocity of the *x* direction, *v* is the horizontal velocity of the *y* direction, and *D_t_* is the time step. The velocity is calculated by the three-dimensional fluid dynamics model.

The wind guide transport is another important factor of advection transport. The direct effect of the wind film transport can be expressed as:

(3) U=f⋅W

where W is the wind speed vector and f is the wind factor matrix. The speed of the wind guide is generally 0.8% to 5.8%, and the declination is between 0° and 45°. According to the perennial predominating wind direction and seasonally predominating wind direction for Changxing Island, combined with the climate features for the maximum probability of the accident, in this model, the speed of the wind guide is 2% of the wind speed, and the declination is approximately 15°. In addition, nonlinear waves generated by wave residual currents (waveguide transport) also contribute as one of the factors of the oil particle span.

(2) Turbulent diffusion process

In recent years, many scholars have adopted the random walk method to simulate turbulent diffusion processes. Within each time step, turbulent diffusion of one oil particle is represented by the following formula:

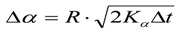
(4)
where Δα is the α direction turbulent diffusion distance ( α = x, y and z), *R* is the normal distribution random number, N(0,1), K*α* is the α direction turbulent diffusion coefficient, and Δt is the time step.

(3) Vertical diffusion process

The random walk method and buoyancy have the same order of magnitude. In addition, the vertical average flow on the movement of the oil droplets also has a specific contribution, but the magnitude is small so it will not be considered in the model. For the vertical diffusion coefficient in the model, we used the formula:

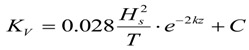
(5)
where *K_V_* is the vertical turbulent diffusivity, *H_s_* is effective wave, *k* is wave number, *T* is wave cycle, *z* is depth, and *C* is constant.

(4) Vertical transport under the action of buoyancy

The calculation formula of probability of the depth that oil dropped into the water is as follows:

(6) $$P=\exp(-{\left(\frac{d}{c}\right)}^{b})$$

where *P* is probability, *c* is characteristic length, *b* is constant, and *d* is oil dropped into the water depth. The experimental result obtained by *b* ≈ 1 assumes that the oil dropped into the water depth has a 5% chance of more than one or two times wave height. The *c* value can be calculated by Equation (6) and the oil drip water depth probability can be determined. Equation (6) showed that the oil droplets into the water concentration are of an exponential distribution. The experiments show that the water depth and wave height of the oil droplets are the same order of magnitude as maximum wave height.

Van Eldik proposed that oil spillage will be linked to the water with wave elements using the following empirical formula, it is ratio between the volume of oil spill into the water and the initial volume:

(7) $$\frac{V_{e}}{V_{0}}=1-e^{C_{2}\cdot t\cdot H_{d}^{2}/L}$$

Where $C_{2}=-2.53\times 10^{-3}/V_{0}^{0.62}$ , *V_e_* is the volume of oil spill into the water, V_0_ is the initial volume, *H*_d_ is the significant wave height, *L* represents the wavelength, and t is the time. Another calculation formula of oil spill into the water is expressed as a function of wind conditions: F = C (W + 1)^2 ^where W represents winds, and C represents the into water coefficient. The water probability is given as:

(8) $$P_{s}=1-\exp(-F\Delta t)$$

Every time the oil droplets enter the water, a certain distribution of diameter is randomly chosen. The desired size distribution of the water droplets is a normal distribution. Delvigne’s experiments also show that the type of droplet size distribution of the vertical diffusion of light crude oil under natural conditions is close to a normal distribution. The normal distribution will be used in this system; the average diameter of oil drops is 250 µm, and the square deviation is 75 µm.

The coefficient of water will directly influence the water entry rate coefficient. By numerical experiments, the coefficient of water was identified as 3.0 × 10^−5^. The floatation process is the distinction between large particles and small oil drops below the critical diameter:

(9) $$d_{c}=\frac{9.52v^{\frac{2}{3}}}{g^{\frac{1}{3}}\cdot {\left(1-\rho_{0}/\rho_{w}\right)}^{\frac{1}{3}}}$$

where v is the kinematical viscosity of sea water, $\rho_{0}$ is oil density, and $\rho_{w}$ is sea water density. For small oil droplets $d_{i}<d_{c}$ ), the rising velocity is given by Stokes law:

(10) $$U_{LS}=g{d_{i}}^{2}(1-\rho_{0}/\rho_{w})/(18\cdot v)$$

For big oil droplets ( $d_{i}\;\geq \;d_{c}$ ), the rising velocity is given by Reynolds law:

(11) $$U_{LS}=\sqrt{\frac{8}{3}\cdot }gd_{i}(1-\rho_{0}/\rho_{w})$$

(5) The oil particle diffusion boundary conditions

In the model, the oil particle cloud movement is three-dimensional; oil viscosity is bigger and the treatment of oil particles reaches the surface and bottom with no reflective condition. When the water oil droplets rise to the surface, they become surface particles, and together with the other surface particles, they flow over the surface and spread. These surface particles then enter the water again by wave action. When the water droplets fall and remain on the bottom of the sea, they are considered “dead” in the simulation and are not included in the calculations. Oil particles may reach land in the drift process. We also considered these particles to be “dead” in the simulation and did not include them in the calculation. Ocean and coastal boundary treatment has limitations, as bottom or landing oil could be under the action of turbulence. A quantitative description of these processes is not reasonable; therefore, this model adopts the above processing method.

(6) Changing properties of oil spillage

The surface of an oil spillage also experiences evaporation and emulsification due to various weather processes in the transport and diffusion process, and this will directly lead to changes in the physicochemical properties of the oil film. The oil spillage weathering process is quite complex. This model is mainly used to study the oil spill evaporation and emulsification process; biological degradation and oxidation processes are not considered. We adopt the Stiver and Mackay parameter formula to calculate the rate of evaporation. The evaporation coefficient is defined as:

(12) $$\theta^{\prime }=\frac{K\prime At}{V_{0}}=\frac{k\prime t}{\delta }$$

where $k^{\prime }=2.5\times 10^{-3}U_{w}^{0.78}$ , *U_w_* is the wind speed 10 m above the ocean surface, *A* is the area of oil film, $V_{0}$ is the overflow oil of the beginning physical volume, and *t* is time. The evaporation rate is a function of the evaporation coefficient, the boiling point temperature and other factors:

(13) $$F_{V}=\ln\left[[1+B\prime (\frac{T_{G}}{T})\theta \prime \exp\left(A^{\prime }-B^{\prime }\frac{T_{0}}{T}\right)\right]\frac{T}{B\prime +T_{G}}$$

where *F_V_* is an evaporation rate (A′ = 6.3, B′ = 10.3), *T_G_* is the gradient of boiling point curve, *T* is the oil temperature, and *T_0_* is the oil (with $F_{V}$ = 0) beginning boiling point temperature.

The emulsification process is affected by wind, wave action, thickness of the oil and ambient temperature factors. Generally, we can use moisture (*Y_W_*) to characterize the degree of emulsification, computing the emulsion moisture formula as follows:

(14) $$Y_{W}＝\frac{1}{K_{B}}(1-e^{-K_{A}K_{B}{\left(1+U_{W}\right)}^{2}}t)$$

where $K_{A}$ = 4.5 × 10−6, *U_W_* is the wind speed, $K_{B}=\frac{1}{Y_{W}^{F}}\approx 1.25$ , $Y_{W}^{F}$ is the final moisture content, and *t* is time.

The effect of emulsification on oil density is expressed as: $\rho_{e}=\left(1-Y_{W}\right)\rho_{0}+Y_{W}\rho_{W}$ , where $\rho_{e}$ is the density of the emulsified oil, $\rho_{0}$ is the emulsified oil before initial density, $\rho_{w}$ is seawater density, and *Y_W_* is the moisture content of the emulsified material. The evaporation of the oil density is expressed as: $\rho =\left(0.6\cdot \rho_{0}-0.34\right)\times F_{V}+\rho_{0}$ .

With comprehensive influences of the two, the density of the oil is expressed as:

(15) $$\rho =(1-Y_{W})[(0.6\times \rho_{0}-0.34)\times F_{V}+\rho_{0}]+Y_{W}\times \rho_{W}$$

The oil viscosity changes with temperature; this model assumes that the temperature change is small so it does not consider the effect of temperature on the viscosity. Changes in oil viscosity are mainly the effect of both emulsification and evaporation.

Emulsified oil viscosity will increase: $v_{e}=v\cdot exp(2.5Y_{W}/(1-0.654Y_{W}))$ , where *V_e_* is the kinematic viscosity of the emulsified oil, *v* is kinematic viscosity before emulsification, and *Y_W_* is the water content of the emulsion. The evaporation has influence on viscosity: $v=v_{0}\cdot 10^{4F_{v}}$ , where *v_0_* is the movement of oil using the initial viscosity coefficient. 

Integrating the two effects, the oil viscosity expression is:

(16) $$v=v_{0}\cdot 10^{4F_{v}}\cdot \exp(2.5Y_{w}/(1-0.654Y_{w}))$$

### 2.2. Research Techniques

(1) Calculation of regional choice

We used the waters around Changxing Island for the purposes of this model. 

(2) Oil spillage quantity and determination of overflow location

Between 1973 and 2006, China’s major oil spill accidents accounted for more than 1000 t; the average oil content was 2701 t. Oil spill risks of 2700 t and greater are predicted in this model. We selected the western channel into the Koumen as the spill site.

(3) The selection of weather conditions

The surrounding sea area is affected by monsoons, southern winds in the summer and northern winds in the winter. According to the historical observations, combining the characteristics of oil spillage impact prediction with the distribution characteristics of environmental sensitive areas nearby. We determined the following situations as the weather conditions for our dynamic oil spillage drift prediction:

- Calm wind: the static wind frequency is 7.79%.
- Northerly wind: NNE direction, the static wind frequency is 18.25%, wind speed of 10 m/s.
- Westerly wind: WSW direction, the static wind frequency is 13.68%, wind speed of 10 m/s.
- Southerly wind: SSW direction, the static wind frequency is 26.45%, wind speed of 10 m/s.

(4) Oil spillage impact prediction programs

Oil spillage impact predictions are summarized as follows: (i) In the still air, near the Koumen; (ii) SSW wind conditions, near the Koumen; (iii) WSW wind conditions, near the Koumen; and (iiii) SSW wind conditions, near the Koumen. Flows are a condition of conventional dynamic effects involved in forecasting calculation. To simplify the calculation workload, the trend of the influence of the initial phase will not be discussed.

### 2.3. Establishment of Evaluation Index System

There are many factors affecting the oil spillage risk. According to the ITOPF statistical data, following the principles of scientificity, metricability and operability, we build the oil spillage risk assessment comprehensive index system, which includes six influencing factors and twenty-three evaluation indexes. We analyze six risk factors including allision/collision, grounding, hull failure, equipment failure, fire/explosion and other/unknown, and calculate the percentage of accidents caused by these factors (from 1970 to 2013) according to the ITOPF survey data. Meanwhile, we calculate the score of these factors based on the weight of damage effects caused by the factors (the historical maximum damage weight is 100) and establish the evaluation index including four indexes: loading/discharging, bunkering, other operations, and unknown reasons, and calculate the percentage of accidents caused by the risk factors according to the ITOPF survey data (from 1970 to 2013), taking the damage weight into account at the same time, and determining each evaluation index. This determines the weight value based on the contingency occurrence probability and severity of consequence, as shown in Table 1.

**Table 1:** The oil spillage risk assessment index system.

Target Level (A)	System Level(B)			Index Level(C)		
	Risk Influencing Factors	Proportion (%)	Index	Evaluation Index	Proportion (%)	Index
Oil spillage risk assessment	Allision/Collision	7.0	1.46	Loading/Discharging	1.1	0.02
				Bunkering	1.1	0.02
				Other Operations	7.6	0.11
				Unknown	90.2	1.31
	Grounding	6.8	0.15	Loading/Discharging	0.8	0.001
				Other Operations	5.8	0.009
				Unknown	93.4	0.14
	Hull Failure	7.6	5.03	Loading/Discharging	56.3	2.83
				Bunkering	1.7	0.09
				Other Operations	8.2	0.41
				Unknown	33.8	1.70
	Equipment Failure	19.7	2.31	Loading/Discharging	66.9	1.54
				Bunkering	6.2	0.14
				Other Operations	14.9	0.34
				Unknown	12.0	0.29
	Fire/Explosion	2.8	0.08	Loading/Discharging	28.9	0.023
				Bunkering	2.9	0.003
				Other Operations	20.2	0.016
				Unknown	48.0	0.038
Oil spillage risk assessment	Other/ Unknown	56.1	0.97	Loading/Discharging	33.2	0.32
				Bunkering	8.9	0.09
				Other Operations	18.5	0.18
				Unknown	39.4	0.38

By reference to the ecological safety assessment risk level division standard, adopting the non-equidistance method [20], the value range from 0 to 10 of risk assessment comprehensive index by calculation is divided into five different level intervals, as shown in Table 2.

**Table 2:** Risk ranking of oil spillage.

Risk level division	Lowest	Lower	Medium	Higher	Highest
Risk assessment index (R)	R < 4.0	4.0 ≤ R < 6.0	6.0 ≤ R < 7.0	7.0 ≤ R < 9.0	9.0 ≤ R

## 3. Results and Discussion

### 3.1. Prediction Results of Oil Spill Effects Near the Koumen

(1) Windless conditions 

**Figure 1:**
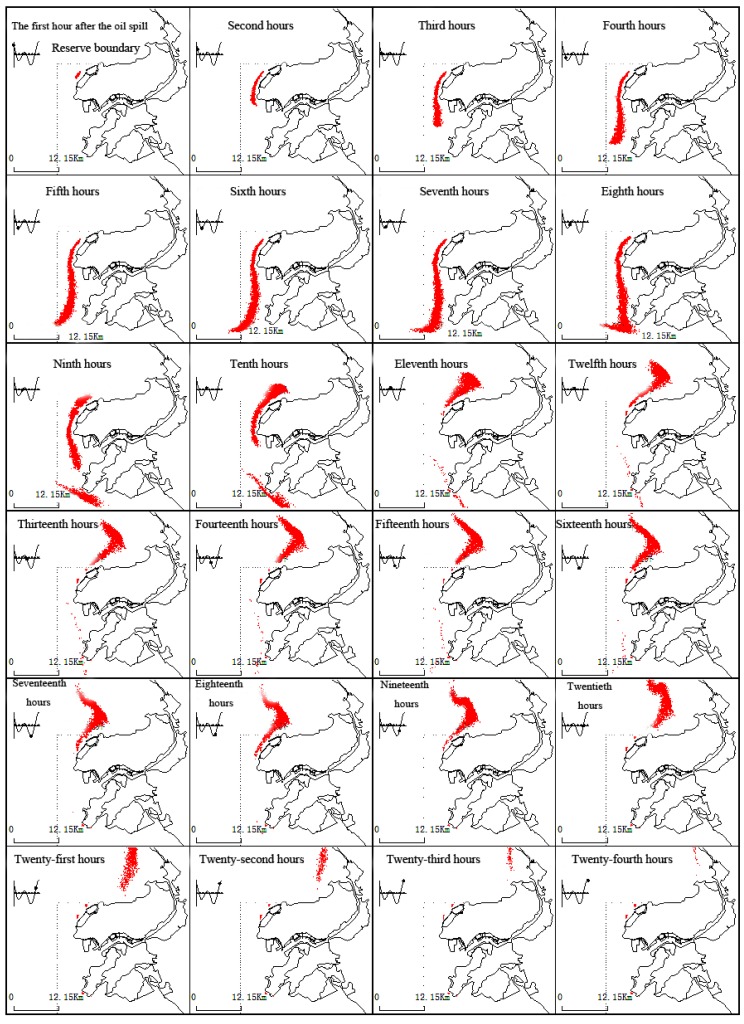
The dynamic changes of the oil film under continuous drift tide conditions.

Figure 1 shows oil spillage near the Koumen in a continuous, 24 h drift process. After oil is spilled, under the action of ebb currents, it drifts southward. After hour six the oil gradually drifts south out of the calculation area. Flooding began at hour eight, and oil spillage under the action of flood currents drifted to the north. At 19 h, part of the oil spillage drifted out gradually from the north. In this process, a large portion of oil film is under flood drivers and drifted into the northeast Fuzhou Bay; it is possible that this process could also cause oil to land on the shores of the Xianyu Bay area and to enter harbor seal core habitat area in the nature reserves.

(2) NNE wind conditions

**Figure 2:**
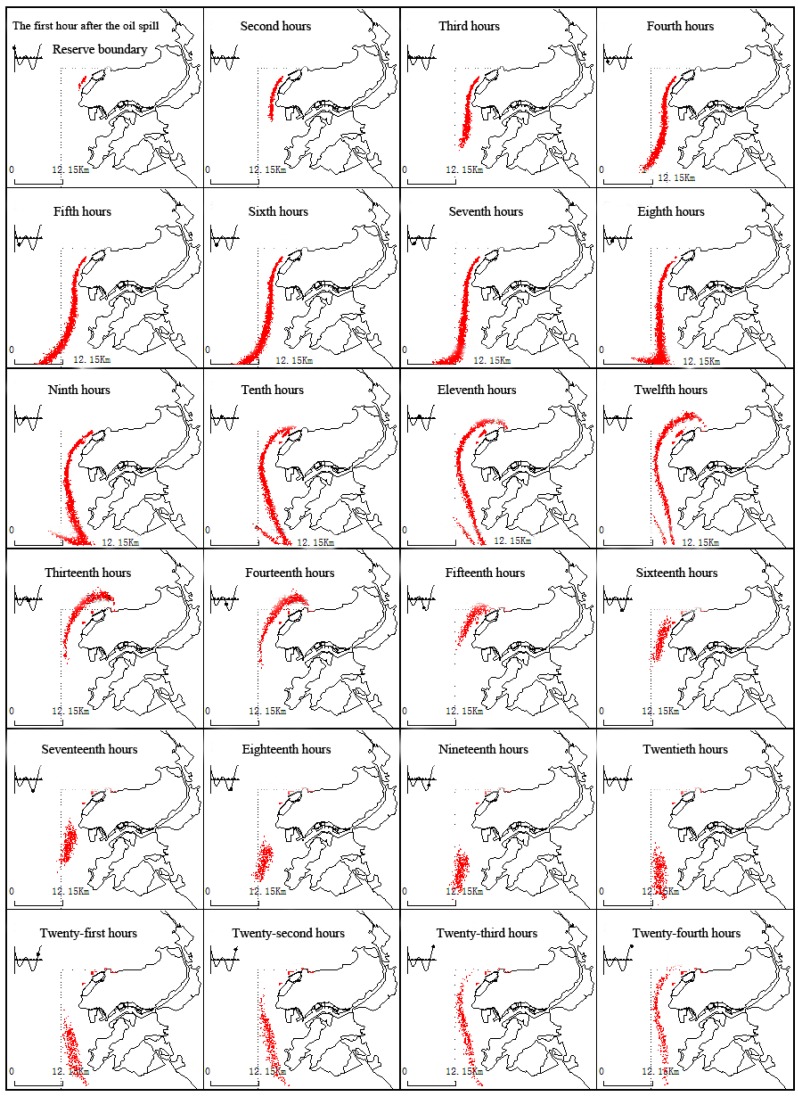
The dynamic changes of the oil film under continuous drift and NNE wind conditions and tide.

Figure 2 presents oil spillage drift in NNE winds and tidal currents near the Koumen. In this case, the oil spillage drifts to the south, and after the fifth hour, the oil gradually drifts south out of the boundary area. In this drift process, part of the oil film will land on the north port breakwater.

(3) SSW wind conditions

**Figure 3:**
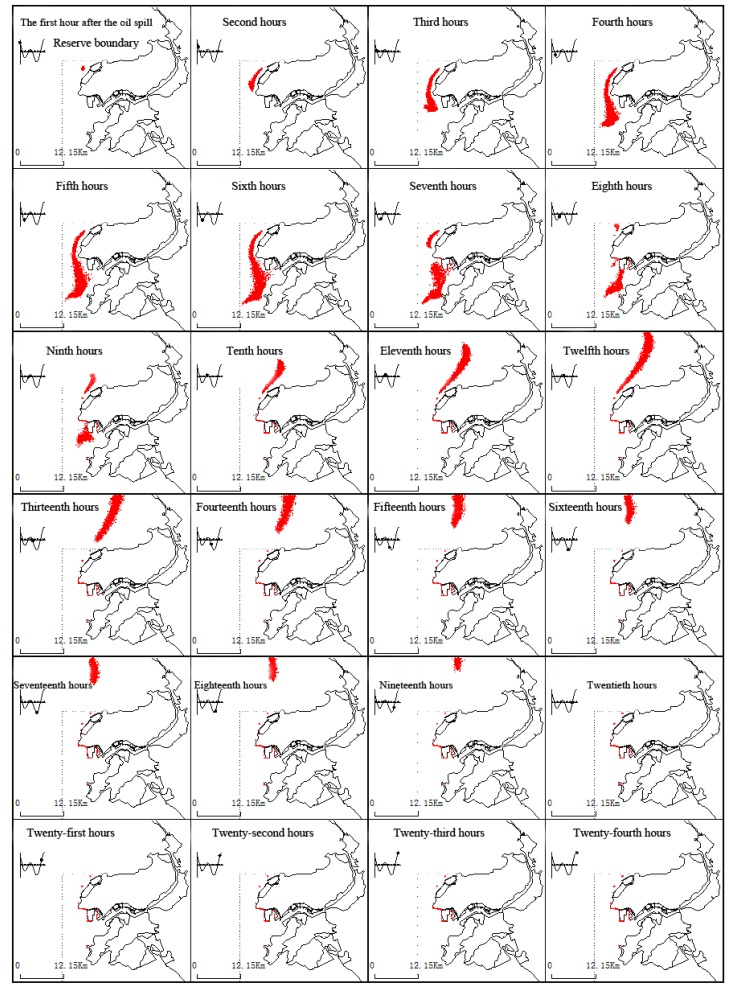
The dynamic changes of the oil film under continuous drift and SSW wind conditions and tide.

Figure 3 presents the drifting and spreading processes near the Koumen during SSW winds and tides. In this case, the oil film drifts along the west coast of Changxing Island southward.

After the third hour, a portion of the oil film enters Hulushan Bay and may land on the north and south shores. After the eighth hour, another portion of the oil film drifts northeast into Fuzhou Bay area under flood action. After the twelfth hour, the oil film exits the northern boundary of the calculation.

(4) WSW wind conditions

**Figure 4:**
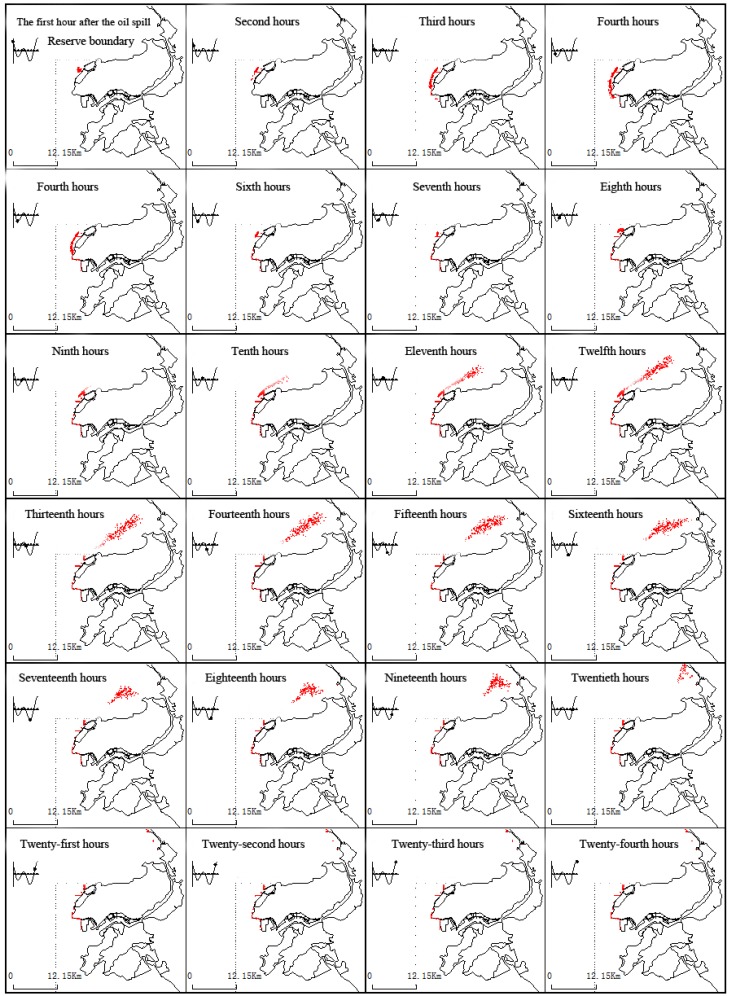
The dynamic changes of the oil film under continuous drift and WSW wind conditions and tide.

Figure 4 presents the expansion process near the Koumen with the presence of WSW winds and tides. Initially, the oil film drifts southwest along the coast and is driven by tidal ebb flows and WSW winds; a portion of the oil moves to the northern outer edges. After the ninth hour, the oil film drifts northeast into the Fuzhou Bay. After the sixteenth hour, the oil film lands on the beaches of Xian Yu Bay.

### 3.2. Environmental Risk Assessment Conclusions 

#### 3.2.1. Prediction Results of Oil Spillage Risk Level 

In accordance with the investigation, there exist oil spillage risks such as collisions, groundings, hull damage, equipment trouble, and fire/explosion of the Changxing Island sea area in Dalian. Some are caused by loading and unloading, refueling and other operations, some are not known. According to the survey, we calculate the percentage of factors such as loading/discharging, bunkering, other operations, and unknown reasons in all the risk factors, and contrast with the index weights in Table 1. On the base of the index in Table 1, we determine the weights of each index of this area, and accumulate these indexes. In the end the calculated evaluation index R is 6.12. Adopting the established oil spillage risk assessment comprehensive index system to evaluate the oil spill accident risk of Changxing Island in Dalian, on the basis of evaluation standard in Table 2, the oil spillage risk level in this area is medium.

#### 3.2.2. The Sphere of Influence of a Potential Oil Spillage

The results of our numerical simulation of oil spillage risk demonstrates the necessity of major planning in the northern Changxing Island ports. Once the oil spillage occurred, the drift expanded under the action of wind and tidal currents. The main influence was sea conditions with no wind (or breeze); in this case the oil spillage affected the waters to the west and northwest of Changxing Island. During the flood tide, the oil spillage will enter the core area of the harbor seal nature reserve. The oil spillage drifts southwest with NNE winds and the trend of the conditions and influences the ecological environment west of Changxing Island. Under SSW wind and current conditions, the oil spillage enters the harbor seal core habitat area of the nature reserve. During onshore wind conditions, the oil spillage lands on the west coast of Changxing Island.

#### 3.2.3. Oil Spillage Impact on the Harbor Seal

Harbor seals live in the ocean in cool temperature zones, and are listed as first-grade state protection animals. They need to be on ice, beach or rocks when they are reproducing, resting and molting, but at all other times, they swim and feed in the sea, so sea water polluted by oil will have a significant impact on their lives. The core habitat area of Dalian harbor seals is located in the northern Gulf of Changxing Island, as well as an experimental habitat area west of the reserve. The occurrence of an oil spill in the harbor seal nature reserve will directly affect both the leopard seal and the surrounding marine environment under any type of condition.

#### 3.2.4. Analysis of the Effect on the Breeding Area

According to the model, an oil spill under any type of condition will spread to the breeding area and influence the cultivation area.

### 3.3. Oil Spillage Prevention Measures and Emergency Countermeasures 

There is a small probability of an oil spillage event; however, the amount of environmental harm could be enormous. Therefore, it is important to strengthen prevention and early warning research to reduce the probability of an accident. From all aspects of design, construction, safety and environmental management, production management,* etc.*, we take reasonable measures to prevent oil spillage accidents as the most effective way to reduce environmental risk. According to the planning of Changxing Island port, wharfs and berths will be built in the north port area; there is a risk of oil spillage accidents in the construction and operation process of these projects. To prevent the risk of oil spillage, we present the following prevention and emergency measures:

(1) A guarded construction period. 

Each construction company should coordinate a construction period plan for protection and preparation under bad weather conditions (work will cease under strong winds greater than class six to avoid ship accidents). The role of the observation on construction ships should be strengthened and construction personnel should strictly follow procedures. During an emergency, the necessary measures should be taken immediately and reported to the corresponding management departments.

(2) The establishment of oil spillage emergency mechanisms.

There is an increased risk of an oil spill with the Dalian Changxing Island north port construction and expansion. The development of an oil spill emergency command is necessary north of the port, as well as an oil spill emergency response system. There are plans to conduct professional training to strengthen the understanding of emergency control procedures, to master emergency prevention and the operation of the equipment used and to increase the ability to dispose of accidental oil spillage. There are marine oil spill emergency response exercises on a regular basis to simulate the emergency response ability of each department to ultimately reduce water and environmental pollution.

(3) The preparation of an operational oil spill contingency plan.

This draws up a contingency plan according to the characteristics of the region; this emergency plan is the implementation of command procedures to yield an effective response. Emergency plans should include management of the oil spillage, response command, communication and cooperation, risk rates, the scope of influence of the surrounding environment and environmental sensitivity analysis. Accident response may also include indirect factors surrounding environmental recovery, such as filing claims, and public relations coordination.

(4) Oil spill emergency equipment is arranged uniformly according to the requirements on emergency response equipment/facilities for oil spill terminals in ports (Chinese National Standard Number: JT/T451-2009), deployment and use of spillage equipment. 

(5) The establishment of a ship oil spillage emergency surveillance monitoring system.

The emergency monitoring system of marine oil spillage is conducted by monitoring to detect ship oil spillage and other maritime accidents and to quickly determine the location and nature of ship accidents. Monitoring provides the basis for the emergency response measures and programs selected.

## 4. Conclusions

(1) Based on contingency occurrence probability and severity of consequence, the paper establishes an oil spillage risk assessment index system including influencing factors such as collisions, groundings, hull damage, equipment trouble, fire/explosion, determines the weight of each evaluation index, and provides the basis for offshore oil production, transportation safety and management.

(2) The paper evaluated the oil spillage risk in the waters near the island of Changxing in Dalian based on the established risk assessment index. The evaluation results show that the comprehensive risk evaluation index is 6.12, belonging to the medium pollution risk level.

(3) The model prediction results indicate that different meteorological conditions have different effects on Changxing Island when a oil spillage occurs. Under windless conditions, the influenced sea area are western Changxing Island and the northwest sea area. During the flood current conditions, oil spillage will enter the core zone of the harbor seal National Nature Reserve. Under NNE wind and tide conditions, oil spillage would drift south by west and influence the ecological environment of waters west of Changxing Island. Under SSW wind and tide conditions, oil spillage would soon land on the west coast of Changxing Island. Hence, related departments should do good precautionary work combind with the climate characteristics to reduce the probability of occurrence of oil spillage.

(4) By combining index system prediction with model prediction to improve the accuracy of the prediction, through the field investigation, the rationality of the established oil spillage risk comprehensive index system combined with model prediction is further confirmed. Restricted by the data collection and evaluation methods, there remains a degree of uncertainty about the established risk assessment index system and evaluation results, which should be perfected gradually in future research.
